# Clinical Efficacy and Safety of Sodium Thiosulfate in the Treatment of Uremic Pruritus: A Meta-Analysis of Randomized Controlled Trials

**DOI:** 10.3390/toxins13110769

**Published:** 2021-10-30

**Authors:** Ping-Hsun Lu, Hui-En Chuo, Ko-Lin Kuo, Jian-Fu Liao, Po-Hsuan Lu

**Affiliations:** 1 Department of Chinese Medicine, Taipei Tzu Chi Hospital, Buddhist Tzu Chi Medical Foundation, New Taipei City 23142, Taiwan; 101318121@gms.tcu.edu.tw; 2 School of Post-Baccalaureate Chinese Medicine, Tzu Chi University, Hualien 97048, Taiwan; Kl.kuo@tzuchi.com.tw; 3 Department of Dermatology, MacKay Memorial Hospital, Taipei 10449, Taiwan; waynec.4204@mmh.org.tw; 4 Division of Nephrology, Taipei Tzu Chi Hospital, Buddhist Tzu Chi Medical Foundation, New Taipei City 23142, Taiwan; 5 School of Medicine, Buddhist Tzu Chi University, Hualien 97048, Taiwan; 6 Division of Hospitalist Medicine, Taipei Tzu Chi Hospital, Buddhist Tzu Chi Medical Foundation, New Taipei City 23142, Taiwan; tch24228@tzuchi.com.tw; 7 Department of Medicine, MacKay Medical College, New Taipei City 25245, Taiwan

**Keywords:** uremic pruritus, sodium thiosulfate, chronic kidney disease, adverse drug reaction

## Abstract

Uremic pruritus is a distressful complication of chronic kidney disease and results in impaired quality of life and higher mortality rates. Intravenous sodium thiosulfate has been reported to alleviate pruritus in hemodialysis patients. We performed a systematic review and meta-analysis to estimate the efficacy of intravenous sodium thiosulfate in patients with uremic pruritus. A systematic search of electronic databases up to June 2021 was conducted for randomized controlled trials that evaluated the clinical effects of sodium thiosulfate in the management of patients with uremic pruritus. Two reviewers selected eligible articles and evaluated the risk of bias; the results of pruritus assessment and uremic pruritus-related laboratory parameters in selected studies were analyzed. There are four trials published between 2018 and 2021, which include 222 participants. The sodium thiosulfate group displayed significant decrease in the pruritus score (standardized mean difference = −3.52, 95% confidence interval = −5.63 to −1.41, *p* = 0.001), without a significant increase in the adverse effects (risk ratio = 2.44, 95% confidence interval = 0.37 to 15.99, *p* = 0.35) compared to the control group. Administration of sodium thiosulfate is found to be a safe and efficacious complementary therapy in improving uremic pruritus in patients with chronic kidney disease.

## 1. Introduction

Uremic pruritus (UP) affects more than 50% of dialysis patients with advanced chronic kidney disease (CKD) [1]. The presentation of UP varies with the severity, distribution, and duration of pruritus and the timing of onset [2,3]. Adverse medical and psychosocial outcomes have been reported to be associated with UP. Patients with moderate to severe pruritus have a higher risk of mortality, more depressive symptoms, and lower quality of life than those without pruritus. Sleep disturbance due to pruritus may be a crucial cause of increased mortality [3]. The treatments of UP include systemic therapies, topical therapies, and complementary alternative therapies [3]. However, only a few meta-analyses on the efficacy of treatment agents such as gabapentin and nalfurafine exist, which reveal some potential adverse effects of the treatments [4,5]. Therefore, identifying safe and effective complementary therapies for UP is critical.

There are multiple pathophysiologic hypotheses of UP [3]. Previous studies showed that the efficiency of dialysis, metabolism of minerals such as phosphorus and calcium, and parathyroid hormone (PTH) were related to UP [3,6]. However, it is currently thought that serum albumin and inflammatory markers are also related to UP [3,7,8].

Sodium thiosulfate (STS), also called sodium hydrosulfite, is an inorganic agent with antioxidant and vasodilatory properties [9]. STS is indicated for the treatment of acute cyanide poisoning [10]; however, its use for off-label indication has also been reported, such as for calcific uremic arteriolopathy or calciphylaxis, coronary artery calcification [10,11,12,13,14], and cisplatin-induced hearing loss [9,15]. A recent retrospective study proposed the potential therapeutic effect of STS in patients with UP [16]. The possible mechanism of STS in treating UP may result from boosting the production of nitric oxide, which caused vasodilation and reduced local inflammation and pruritogen [3,16].

There is little evidence available regarding the efficacy and safety of STS in UP treatment. We performed a systematic review and meta-analysis of randomized controlled trials (RCTs) to assess the therapeutic and adverse effects of STS in the treatment of UP.

## 2. Results

### 2.1. Study Characteristics

**Figure 1:**
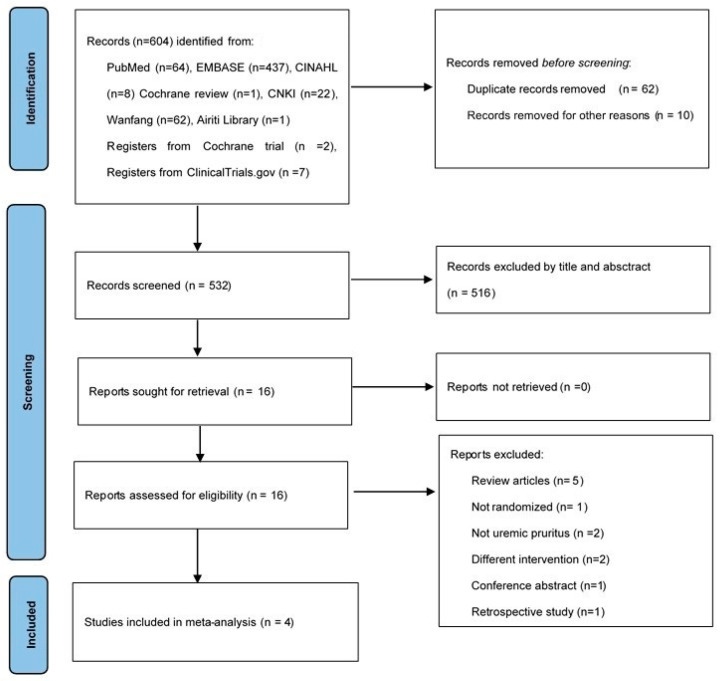
Study flow diagram.

We demonstrate the process of article search and selection in Figure 1. Initially, the search resulted in 604 citations. Seventy-two duplicate articles were removed by Endnote and reviewers. Furthermore, 516 articles were excluded in accordance with the screening criteria for both titles and abstracts. We retrieved the full texts of the remaining 16 articles. After assessing for eligibility, we excluded 12 articles because five were review articles [17,18,19,20,21], one was non-RCT [22], two were not focused on UP [23,24], two were conducted on patients undergoing different treatment interventions [25,26], one was a conference abstract [27], and one was a retrospective study [16]. Finally, four RCTs fulfilled the selection criteria and were included in the meta-analysis [28,29,30,31].

Table 1 exhibits the patient characteristics and demographic data of the included trials. The selected studies were all RCTs released from 2018 to 2021. A total of 222 patients were enrolled in the four studies. The research patients were adults with UP and CKD undergoing hemodialysis (HD). Moreover, subjects were mostly males (65%) with mean ages between 48 and 67 years. In three studies, patients with other skin or systemic diseases were excluded [29,30,31]. Wu et al. and Liu et al. targeted patients with refractory UP in whom first-line therapy failed and who presented with pruritus for >3 months [29,30], whereas Hsu et al. and Xu et al. selected HD patients with any symptoms of pruritus [28,31]. Intravenous STS was administered after the HD session [30,31] or in the last 30 min of HD [28,29] at a frequency of one to three times a week for either 8 weeks [28,29] or 3 months [30,31]. The STS dosage ranged from 0.64 g per 20 mL to 25 g per 100 mL with normal saline. The efficacy of STS was compared to the control group with or without other therapies. The patients of STS groups in Liu et al.’s studies were treated with an oral antihistamine, topical emollient, Chinese herb bath therapy, and acupuncture, and those in Wu et al.’s studies were treated with oral loratadine 10mg daily [29,30]. All RCTs reported the itching scores for UP; including the modified Duo’s pruritus score (mDuo) [28,30], visual analog scale (VAS) [29,30], and Dirk R Kuypers scale [31]. Some selected studies provided information on laboratory data [29,30,31], effective rate [29,30], and adverse drug reactions (ADR) [28,29,30] (Appendix A, Table A1). Liu et al. assessed the effective rate using the following criteria: (1) Highly effective: VAS declined by ≥75% without itching or with mild itching, (2) Effective: VAS declined by 50–75% with obvious improvement in itching, and (3) Ineffective: VAS declined by <50% with severe intolerable itching [29]. Similarly, Wu et al. defined the effective rate using the following criteria: (1) Highly effective: VAS ≤2 points or VAS declined by five points from baseline, (2) Effective: VAS decline by 2–4 points from baseline, and (3) Ineffective: VAS increased or decreased by ≤1 point from baseline [30]. Additionally, the Pittsburgh Sleep Quality Index (PSQI) was used to assess the impact on pruritus-related quality of life [30,31]. PSQI is a self-reported questionnaire that evaluates sleep quality in the past month. It is composed of the following seven component scores: subjective sleep quality, sleep latency, sleep duration, habitual sleep efficiency, sleep disturbance, use of sleep medication, and daytime dysfunction. The sum score of PSQI ranges from 0 to 21 [32].

**Figure 2:**
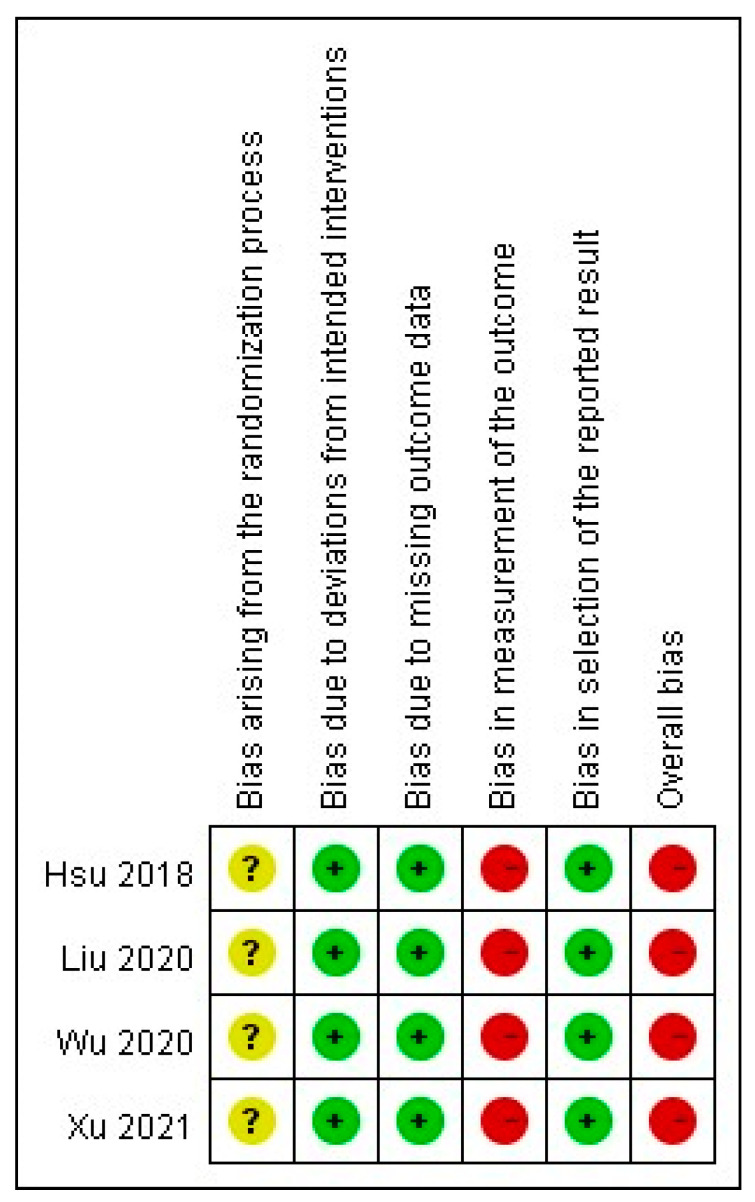
Risk of bias in selected studies.

Figure 2 describes the risk of bias in the selected articles. The methodologic quality of the four RCTs was assessed. Two studies used a random number table to perform the randomization [29,31]; however, the method of allocation concealment was not documented in any of the trials.

One article lacked the baseline data of laboratory parameters [29]. The blinding of patients and assessors was not described in all studies. Moreover, one study lacked the *p*-values for baseline data [29]. The measurement result of pruritus was a participant-reported outcome in all the trials. The selected studies based their analyses on the intention-to-treat principle with an acceptable loss to follow-up rate (<20%). No patients dropped out from the included trials due to ADRs. All outcome data in the studies were reported and analysed.

### 2.2. Outcomes

#### 2.2.1. Pruritus Score

**Figure 3:**
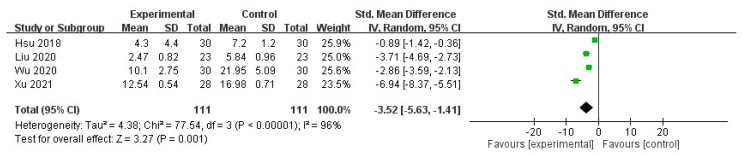
Forest plot for comparison of pruritus scores in UP patients treated with intravenous STS.

The meta-analysis of the selected trials revealed a significant decrease in the pruritus score after intravenous STS administration (mean difference (MD) = −3.52, 95% confidence interval (CI) = −5.63 to −1.41, *p* = 0.001), with significant heterogeneity in the outcome among the studies (I^2^ = 96%, *p* < 0.00001; Figure 3). 

**Figure 4:**
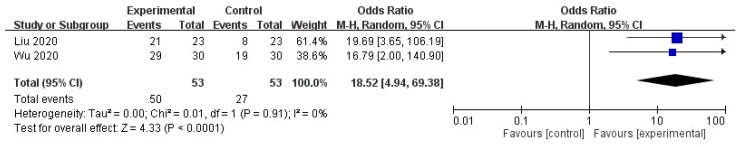
Forest plot for comparison of effective rate in UP patients administered intravenous STS.

**Figure 5:**
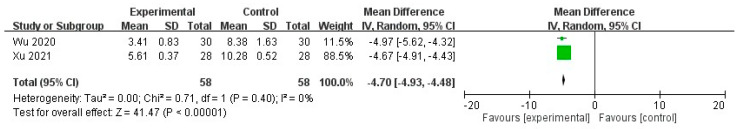
Forest plot for comparison of PSQI in UP patients treated with intravenous STS.

Additionally, subgroup analyses revealed that the effective rate was significantly higher in the STS group than in the control group (odds ratio (OR) = 18.52, 95% CI = 4.94 to 69.38, *p* < 0.0001), with no heterogeneity in the outcome across the studies (I^2^ = 0%, *p* = 0.91; Figure 4). Furthermore, PSQI significantly decreased in the STS group (MD = −4.70, 95% CI = −4.93 to −4.48, *p* < 0.00001), with no heterogeneity in the outcome among the studies (I^2^ = 0%, *p* = 0.40; Figure 5). 

#### 2.2.2. Adverse Drug Reaction

**Figure 6:**
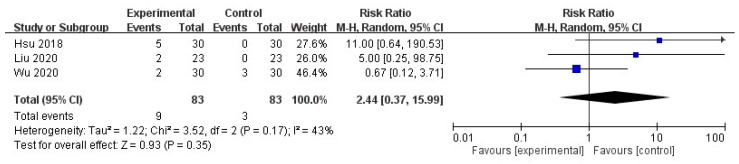
Forest plot for comparison of the number of ADR in UP patients with intravenous STS.

No significant increase was noted in ADRs after intravenous STS administration in patients with UP (risk ratio = 2.44, 95% CI = 0.37 to 15.99, *p* = 0.35), with moderate heterogeneity in the outcome among the studies (I^2^ = 43%, *p* = 0.17; Figure 6).

#### 2.2.3. Laboratory Parameters

**Figure 7:**
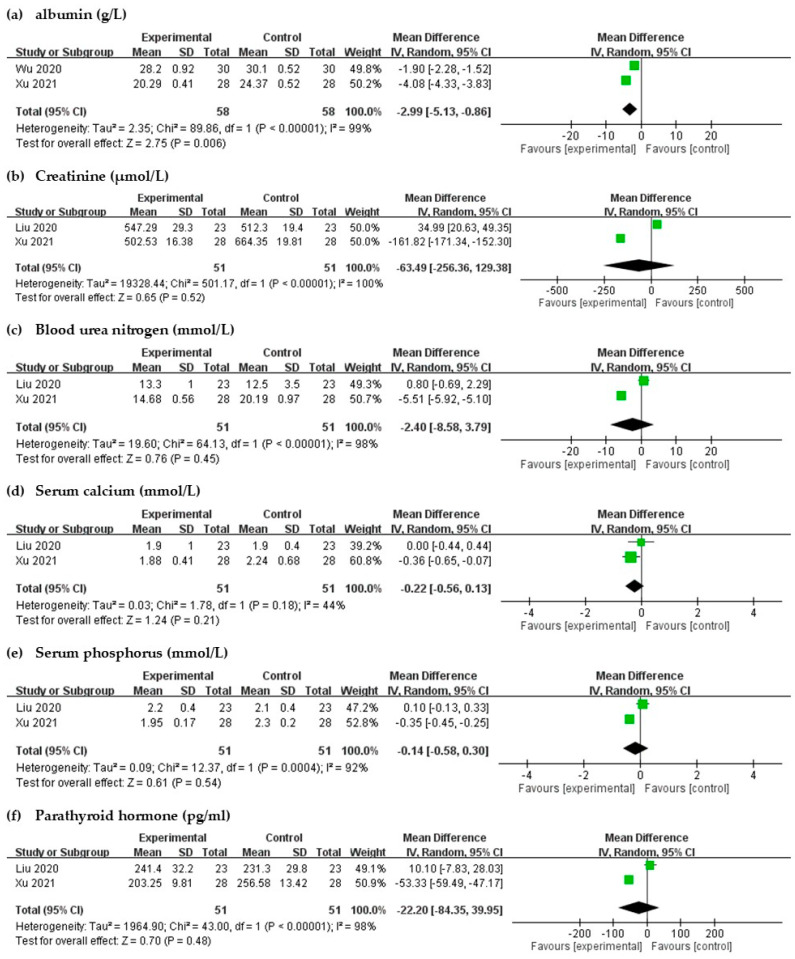
Forest plot for comparison of the laboratory parameters in UP patients with intravenous STS.

Significant differences were noted in the albumin levels after intravenous STS administration (MD = 2.99, 95% CI = −5.13 to −0.86, *p* = 0.006) in laboratory results with high heterogeneity (I^2^ = 99%, *p* < 0.00001, Figure 7). Changes in serum creatinine, blood urea nitrogen, Ca, phosphorus, and PTH were not found to be significant (*p* > 0.05 for each, Figure 7); moderate to high heterogeneities were observed. 

### 2.3. Quality of Evidence

The quality of evidence was very low for STS administration in UP patients. Although the included studies were all RCTs, the risk of bias in randomization and measurement of outcome was serious (Table S1).

## 3. Discussion

### 3.1. Summary of the Meta-Analysis

Our meta-analysis exhibited that administration of intravenous STS significantly reduced the pruritus scores in HD patients; however, ADRs did not increase significantly in the STS group than in the control group.

### 3.2. UP Treatment

Oral antihistamines have been administered as the first-line treatment of UP, despite their reported low efficacy [33]. Patients with UP for whom conventional treatment fails have few effective pharmaceutical choices. Four meta-analyses on the treatment of UP have been published. Wikström et al. pooled two RCTs on nalfurafine, showing better improvement in the VAS scores than the placebo [5]. Another meta-analysis found that gabapentin significantly lowered the severity of UP [4]. Common adverse effects associated with gabapentin and nalfurafine are insomnia, dizziness, headache, and nausea [4,33]. As for alternative medicines, the first meta-analysis on acupuncture or acupressure for UP provided insufficient evidence supporting the use of acupuncture or acupressure [34]. A meta-analysis on Chinese herbal bath therapy showed benefit to patients with UP; however, the efficacy of different herbal bath dosages was unknown [35]. The above treatments of UP caused either potential ADRs or uncertain therapeutic effects [4,5,34,35]. One retrospective study compared the efficacy of intravenous STS administration versus oral 10 mg loratadine daily for 8 weeks in 44 HD patients, and the VAS and detailed pruritus scores in the STS group significantly decreased [16]. The antioxidant or vasodilatory properties related to relieving pruritus in STS remain unclear [16]. We found that the STS group showed significant improvements in not only the pruritus score but also PSQI and effective rates. Itching results in delays in falling asleep and sleep interruption, causing fatigue and decrement in the patients’ quality of life [36]. Approximately 72% of patients with UP are moderately to extremely afflicted by sleep disturbance, which reduces their quality of life and increases their mortality risk [3]. Our study suggested that intravenous STS administration could reduce the severity of pruritus and improve the patients’ sleep quality.

### 3.3. Laboratory Parameters in UP Patient

Previous studies have revealed that UP is related to abnormal metabolism of minerals, such as higher serum Ca, phosphorus, and ferritin levels, and PTH [37,38,39,40]. However, a recent study has shown conflicting results [3]. Rayner et al. reported that older age, higher levels of C-reactive protein, lower serum albumin, and hepatitis B or C infection were more likely to cause itchy skin, and UP was not associated with serum phosphorus, Ca, PTH, Kt/V, and hemodiafiltration [8]. Ozen et al. also stated that white blood cell counts, but not markers of mineral metabolism, were associated with the development of UP [7]. Our meta-analysis did not find a significant difference in serum creatinine, blood urea nitrogen, Ca, phosphorus, and PTH levels, which is consistent with the results of recent studies. The albumin level reflects the nutrition status, suggesting a relation between UP and malnutrition–inflammation complex syndrome [8]. The influence of inflammation and pro-inflammatory factors in patients with UP needs to be investigated in further studies.

### 3.4. Adverse Drug Reaction

The ADRs are common in patients treated with intravenous STS for vascular calcification, calciphylaxis, and cisplatin-induced hearing loss [9,10,13,14,15]. Djuric et al. documented nausea and vomiting (30.4%) and metabolic acidosis (14%) while treating advanced CKD patients of vascular calcification with STS [10]. Furthermore, Udomkarnjananun et al. noted ADRs such as high anion gap metabolic acidosis (32.3%), hypernatremia (18.8%), and nausea and vomiting (24.7%) in patients who were treated with intravenous STS for calciphylaxis [14]. The most common adverse effect of STS during the treatment of cisplatin-induced hearing loss is nausea [15]. One child developed metabolic acidosis during infusion of STS and recovered after discontinuing the treatment [9]. However, in one retrospective study, Song et al. observed that only one patient had temporary palpitations after intravenous STS administration for UP [16]. Our meta-analysis showed that the ADRs, including mild gastrointestinal symptoms (5/83, 6%) [28,30], transient cold symptoms (1/83, 1.2%) [29], chest distress (1/83, 1.2%) [29], and hypotension (2/83, 2.4%) [28,30], did not significantly increase between the STS and control groups, and the percentage of ADRs are lower than aforementioned studies. Moreover, we did not notice any severe adverse effects.

### 3.5. Heterogeneity

The four studies selected in our analysis exhibited specific heterogeneity due to the following factors:

#### 3.5.1. Dosage of STS

The half-life of STS is about 15 min in the serum of patients with intact renal function; however, the mechanism of STS effects in patients under HD is unknown [41]. Therefore, off-label use of STS varies with different doses. Yerram et al. reported that a patient with nephrogenic systemic fibrosis who was accidentally managed with intravenous 12.5 g STS after every HD [42]. Additionally, Brock et al. showed that intravenous STS at a dose of 20 g/m^2^ of body-surface area given 6 h after cisplatin chemotherapy reduced the incidence of cisplatin-induced hearing loss [9]. Several studies have investigated the safety and efficacy of STS on calciphylaxis in patients with CKD [10,11,12,14,41]. The typical dose of intravenous STS is 25 g in 100 mL of normal saline given during the last 30–60 min of HD triweekly [11]. Furthermore, Saengpanit et al. found that intravenous STS at a dose of 12.5 g twice weekly for 6 months during the last hour of HD could significantly reduce the cardio-ankle vascular index [13]. For UP, in a recent retrospective study, intravenous STS at a dose of 3.2 g in 20 mL of normal saline was administered three times per week, which significantly improved UP in HD patients [16]. The dosage and treatment duration of STS in our studies were not consistent across the included studies, making it difficult to conduct simple and straightforward comparisons.

#### 3.5.2. Outcome Measurements

The outcomes were measured at different times and using different tools. The standard pruritus measurement tools for UP are the numerical rating scale, VAS, Duo pruritus score, and 5-D itch scale [3]. The studies included in our meta-analysis used different tools for pruritus assessment, which may explain the high heterogeneity reported. Therefore, the transformation among these diverse pruritus measurement tools is required to analyze the severity and therapeutic effects in different departments or studies. Lia et al. proposed the categories for transformation among the 5-D itch scale and numeric rating scale according to the severity of itching in HD patients [43]. Park et al. used the mapping model to successfully transform the pruritus assessment tool of VAS and EuroQol 5-dimension 3-level utility index [44]. Both the transformation models can be considered as references when conducting related studies in the future. One Cochrane review on chronic pruritus of unknown origin included studies with different assessment tools to measure the same outcome, using standardized mean differences (SMDs) with 95% CIs to analyze the continuous data [45]. Therefore, we performed data synthesis and analysis with SMDs to analyze different pruritus scores in our study. Furthermore, we underwent a subgroup analysis of the uni- and multidimensional scales, including effective rate and PSQI.

#### 3.5.3. Patient Selection

Differences in the severity of pruritus and renal function among the patients may also lead to variable outcomes. The baseline mDuo scores of patients reported in Wu et al.’s study were much higher than those reported in Hsu et al.’s [28,30]; since patients with refractory UP usually presented with higher mDuo scores, the variation in the baseline severity of pruritus may contribute the heterogenicity.

#### 3.5.4. Intervention

Some dissimilarities exist in the interventions among the included trials. Liu et al. treated both groups with a combination of therapies and effective HD [29]. All the patients in Wu et al.’s study received an oral antihistamine daily [30]. These studies included patients who had already received medical therapy for 2 to 6 months [29,30]. This difference among the selected trials caused heterogeneity. Given that UP is usually presented as chronic and refractory pruritus, the findings of our meta-analysis could be applicative.

### 3.6. Limitations

There are certain limitations to our study. First, the methodological flaws involved a high risk of bias in all selected RCTs. Two studies performed the randomization only by using a random number table [29,31]. No trial reported double-blinding. Second, the sample size of the included RCTs was relatively small, and all of them were single-center studies.

## 4. Conclusions

In summary, this meta-analysis on the clinical effect of STS administration against pruritus in patients with UP revealed significant improvement in the patients without remarkable adverse effects. Further investigation with larger sample sizes and well-designed trials focusing on the dosage, frequency, and long-term effects of STS are warranted.

## 5. Materials and Methods

### 5.1. Literature Search

We searched for articles published before June 2021 from the PubMed, EMBASE, CINAHL, Cochrane Library databases, China National Knowledge Infrastructure, Airiti Library, and Wanfang database. The search keywords of MeSH and Emtree were listed: chronic kidney disease OR kidney injury OR kidney failure OR chronic renal failure OR chronic kidney disease OR end-stage renal disease OR dialysis OR hemodialysis OR peritoneal dialysis OR uremic OR Uremia OR uremias, pruritus OR pruritus OR Pruritus OR itch OR xerosis OR skin problems OR skin disorders, and Sodium thiosulfate OR sodium thiosulphate OR sodium hyposulfite OR Hyposulphite of soda OR thiosulfuric acid OR disodium salt. Moreover, we also searched the combinations and free-text words of the terms above and extended the search using the “related articles” function in PubMed. Furthermore, all the retrieved abstracts, studies, and citations were reviewed. Finally, unpublished studies were obtained from the ClinicalTrials.gov registry (http://clinicaltrials.gov/, accessed on 22 June 2021). There were no language restrictions in the search. This meta-analysis was confirmed by the online PROSPERO international prospective register of systematic reviews of the National Institute for Health Research. The search protocol is attached in Supplementary Materials (Table S2).

### 5.2. Study Selection

RCTs were chosen to assess the efficacy of intravenous STS in the treatment of patients with UP. The inclusion criteria were listed below: kidney disease under dialysis, presence of UP, administration of intravenous STS, and itching severity with quantitative data. We removed articles that had not been randomized or had different interventions. If necessary, we would contact the authors of the studies for the original or missing data. For studies with overlapping data, we selected those with a larger population to exclude duplicate articles.

### 5.3. Data Extraction and Quality Assessment

Two reviewers screened the articles and abstracted the information below independently: first author, publication date, participants characteristics, design of the study, inclusion, exclusion, and matching criteria, STS treatment, and quantified data of the severity of itching. According to the inclusion criteria, two reviewers assessed the chosen articles for eligibility. Comments of the two reviewers were recorded and compared. Any disagreements were submitted and reviewed by a third investigator. Furthermore, we performed a quality assessment of the studies using the “risk of bias” tool recommended by the Cochrane Collaboration. Several domains were evaluated: for example, method of allocation, blinding of participants and investigators, the integrity of outcome data, selective reporting, and other kinds of bias.

### 5.4. Data Synthesis and Analysis

We presented outcomes of the following tools for pruritus assessment to evaluate the efficacy of STS: VAS (from no itch [34] to very severe itch [9]), the mDuo (0–40 points), and Dirk R Kuypers score (27 points). The statistical package Review Manager, (Version 5, Cochrane Collaboration, Oxford, England) was applied for data analysis. A meta-analysis was conducted based on recommendations outlined in the PRISMA guidelines. Standard deviations were measured from the provided CI limits, standard errors, or range values when necessary. As the summary statistics, dichotomous outcomes of ADRs were analyzed using risk ratio, and effect estimates were analyzed using the OR. We analyzed and obtained the mean and standard deviation from the studies using the MD or SMD with 95% CI for continuous outcomes. The random-effects model was applied to pool estimates of standard deviation and SMDs, considering the diversity of pruritus assessment tools and possible heterogeneity across the trials. We considered heterogeneity in the studies by performing the I^2^ test and a null hypothesis test, in which *p* < 0.1 evidenced significant heterogeneity among the outcomes. The Guideline Development Tool (GDT) developed by the GRADE Working Group was applied to assign the quality of evidence [46] (Table S1).

## Figures and Tables

**Table 1:** Characteristics of selected studies.

Study (Year)	Study Design	Inclusion Criteria	Previous Treatment	No. of Patients	Age (Years)	STS Dosage, Route, and Frequency	Combination Therapy	Duration	Pruritus Severity Assessment	Pruritus Score (before→after)	Effective Rate	ADR
Hsu et al. 2018	RCT	HD	LC	T:30	T:57.1 ± 5.2	25 g/100 mL NS, i.v., 2~3 times/w	-	8W	mDuo	T: 19.0(8.3)→4.3(4.4)	-	5/30
				C:30	C:56.2 ± 5.6					C: 18.5(7.7)→7.2(1.2)		0/30
Liu et al. 2020	RCT	HD	TE CHT AC AH	T:23	T:67.0 ± 10.4	3.84 g/20 mL NS i.v., 3 times/w	TE CHT AC AH	8W	VAS	T: 9.12(0.34)→2.47(0.82)	21/23	2/23
				C:23	C:65.5 ± 13.5					C: 9.23(0.16)→5.84(0.96)	8/23	0/23
Wu et al. 2020	RCT	HD	TE	T:30	T:48.14 ±10.58	3.2~5.76 g/20 mL NS i.v., 3 times/w	AH	3M	mDuo	T: 30.80(7.33)→10.10(2.75)	29/30	2/30
				C:30	C:51.22 ± 10.21					C: 31.34(8.40)→21.95(5.09)	19/30	3/30
Xu et al. 2021	RCT	HD	LC	T:28	51.21 ± 4.90	0.64 g/20 mL NS i.v., 1~3 times/w	-	3M	DRKS	T: 20.36(1.63)→12.54(0.54)	-	-
				C:28						C: 20.35(1.60)→16.98(0.71)	-	-

Data are presented as mean (standard deviation). RCT = Randomized Controlled Trial; HD = hemodialysis; T = the STS group S = STS; C = the control group; i.v. = intravenous; NS = normal saline; LC = lifestyle changes; TE = topical emollients; AH = oral antihistamine; CHT = Chinese herb bath therapy; AC = acupuncture; mDuo = modified DUO score; VAS = visual analogue scale; ADR = adverse drug reaction; Dirk R Kuypers scale = DRKS.

## Data Availability

The data presented in this study are available in the article and supplementary material section.

## Supplementary Materials

The following are available online at https://www.mdpi.com/article/10.3390/toxins13110769/s1, Table S1: Grade profile summary of ‘STS for UP’ quality assessment; Table S2: Search protocol.

## Appendix A

**Table A1:** Other outcomes of Selected Studies.

Study (Year)	Effective Rate	ADR	Alb (g/L)	Calcium (mmol/L)	Phosphorus (mmol/L)	Scr (μmol/L)	BUN (mmol/L)	PTH (pg/mL)	PSQI	WHOQOL- 100	CRP	Ferritin
Hsu et al. 2018	--	5/30 0/30	--	--	--	--	--	--	--	--	--	--
Wu et al. 2020	29/30 19/30	2/30 3/30	T:25.3 (1.50) →28.2 (0.92) C:28.4 (0.69) →30.1 (0.52)	--	--	--	--	--	T:18.25 (2.30)→3.41 (0.83) C:17.96 (1.90)→8.38 (1.63)	--	T:8.20 (0.59) →6.30 (0.35) C:9.20 (0.87) →7.60 (0.42)	T:509.30 (13.55)→486.52 (10.31) C:508.77 (11.59)→489.42 (8.59)
Liu et al. 2020	21/23 8/23	2/23 0/23	--	T: 1.9 (1.0) C:1.9 (0.4)	T: 2.2 (0.4) C: 2.1 (0.4)	T: 547.9 (29.3) C: 512.3 (19.4)	T: 13.3 (1.0) C: 12.5 (3.5)	T: 241.4 (32.2) C: 231.3 (29.8)	--	--	--	--
Xu et al. 2021	--	--	T:28.69 (0.84)→20.29 (0.41) C:28.70 (0.85)→24.37 (0.52)	T:2.52 (0.35) →1.88 (0.41) C:2.55 (0.33 )→2.2 4(0.68)	T:2.56 (0.21) →1.95 (0.17) C:2.55 (0.23) →2.30 (0.20)	T:788.97 (22.59)→502.53 (16.38) C:789.01 (22.61)→664.35 (19.81)	T:26.34 (1.50)→14.68 (0.56) C:26.35 (1.52)→20.19 (0.97)	T:300.36 (15.37)→203.25 (9.81) C:300.31 (15.32)→256.58 (13.42)	T:14.35 (2.34)→5.61 (0.37) C:14.36 (2.33)→10.28 (0.52)	T: 56.38 (6.17) →83.59 (2.24) C:56.39 (6.20)→71.36 (3.55)	--	--

ADR = adverse drug reaction; Alb = albumin; BUN = blood urea nitrogen; Ca = calcium; CRP = C-reactive protein; PTH = parathyroid hormone; PSQI = Pittsburgh sleep quality index; Scr = serum creatinine; WHOQOL-100 = the World Health Organization Quality of Life 100. Note: Conversion factors for units: Ca in mg/dL to mmol/L, ×0.2495; Phosphorus in mg/dL to mmol/L, ×0.3229; Scr in mg/dL to μmol/L, ×88.4; BUN in mg/dL to mmol/L, ×0.357.
