# Supplementary material for: Clinical Efficacy and Safety of Sodium Thiosulfate in the Treatment of Uremic Pruritus: A Meta-Analysis of Randomized Controlled Trials
Source: Toxins (Basel). 2021 Oct 30;13(11):769. doi: 10.3390/toxins13110769 (PMC8624535; doi:10.3390/toxins13110769)
Supplement: Supplementary file 1 [file toxins-13-00769-s001.zip › toxins-1427742-supplementary.pdf]

Supplementary Materials: Clinical Efficacy and Safety of Sodium Thiosulfate in the Treatment of Uremic Pruritus: A Meta-Analysis of Randomized Controlled Trials

Ping-Hsun Lu, Hui-En Chuo, Ko-Lin Kuo, Jian-Fu Liao and Po-Hsuan Lu

Table S1. Grade profile summary of 'STS for UP' Quality assessment

Certainty assessment							№ of patients		Effect		Certainty	Importance
№ of studies	Study design	Risk of bias	Inconsistency	Indirectness	Imprecision	Other considerations	Pruritus scores	placebo	Relative (95% CI)	Absolute (95% CI)		
**pruritus scores**												
4	randomised trials	serious	serious	not serious	not serious	publication bias strongly suspected	111	111	-	SMD 3.52 lower (5.63 lower to 1.41 lower)	⊕◯◯◯ VERY LOW	IMPORTANT

Certainty assessment							№ of patients		Effect		Certainty	Importance
№ of studies	Study design	Risk of bias	Inconsistency	Indirectness	Imprecision	Other considerations	PSQI	placebo	Relative (95% CI)	Absolute (95% CI)		
**Pittsburgh sleep quality index**												

Certainty assessment							№ of patients		Effect		Certainty	Importance
№ of studies	Study design	Risk of bias	Inconsistency	Indirectness	Imprecision	Other considerations	Pruritus scores	placebo	Relative (95% CI)	Absolute (95% CI)		
2	randomised trials	serious	not serious	not serious	not serious	publication bias strongly suspected	58	58	-	MD 4.7 lower (4.93 lower to 4.48 lower)	⊕⊕◯◯ LOW	IMPORTANT

Certainty assessment							№ of patients		Effect		Certainty	Importance
№ of studies	Study design	Risk of bias	Inconsistency	Indirectness	Imprecision	Other considerations	Effective rate	placebo	Relative (95% CI)	Absolute (95% CI)		
**Effective rate**												
2	randomised trials	serious	not serious	not serious	not serious	publication bias strongly suspected	50/53 (94.3%)	27/53 (50.9%)	OR 18.52 (4.94 to 69.38)	441 more per 1,000 (from 327 more to 477 more)	⊕⊕◯◯ LOW	IMPORTANT

Certainty assessment							№ of patients		Effect		Certainty	Importance
№ of studies	Study design	Risk of bias	Inconsistency	Indirectness	Imprecision	Other considerations	ADR	placebo	Relative (95% CI)	Absolute (95% CI)		
**Adverse drug reaction**												
3	randomised trials	serious	serious	not serious	serious	publication bias strongly suspected	9/83 (10.8%)	3/83 (3.6%)	RR 2.44 (0.37 to 15.99)	52 more per 1,000 (from 23 fewer to 542 more)	⊕◯◯◯ VERY LOW	IMPORTANT

Certainty assessment							№ of patients		Effect		Certainty	Importance
№ of studies	Study design	Risk of bias	Inconsistency	Indirectness	Imprecision	Other considerations	Lab	placebo	Relative (95% CI)	Absolute (95% CI)		
**Serum creatinine**												
2	randomised trials	not serious	very serious	not serious	serious	publication bias strongly suspected	51	51	-	MD 63.49 lower (256.36 lower to 129.38 higher)	⨁◯◯◯ VERY LOW	IMPORTANT
**Blood urea nitrogen**												

Certainty assessment							№ of patients		Effect		Certainty	Importance
№ of studies	Study design	Risk of bias	Inconsistency	Indirectness	Imprecision	Other considerations	Lab	placebo	Relative (95% CI)	Absolute (95% CI)		
2	randomised trials	not serious	very serious	not serious	serious	publication bias strongly suspected	51	51	-	MD 2.4 lower (8.58 lower to 3.79 higher)	⊕◯◯◯ VERY LOW	IMPORTANT

Albumin

2	randomised trials	not serious	not serious	not serious	serious	publication bias strongly suspected	58	58	-	MD 2.99 lower (5.13 lower to 0.86 lower)	⊕⊕◯◯ LOW	IMPORTANT

Calcium

Certainty assessment							№ of patients		Effect		Certainty	Importance
№ of studies	Study design	Risk of bias	Inconsistency	Indirectness	Imprecision	Other considerations	Lab	placebo	Relative (95% CI)	Absolute (95% CI)		
2	randomised trials	not serious	serious	not serious	serious	publication bias strongly suspected	51	51	-	MD 0.22 lower (0.56 lower to 0.13 higher)	⊕◯◯◯ VERY LOW	IMPORTANT

Phosphorus

2	randomised trials	not serious	serious	not serious	serious	publication bias strongly suspected	51	51	-	MD 0.14 lower (0.58 lower to 0.3 higher)	⊕◯◯◯ VERY LOW	IMPORTANT

Parathyroid hormone

Certainty assessment							№ of patients		Effect		Certainty	Importance
№ of studies	Study design	Risk of bias	Inconsistency	Indirectness	Imprecision	Other considerations	Lab	placebo	Relative (95% CI)	Absolute (95% CI)		
2	randomised trials	not serious	serious	not serious	serious	publication bias strongly suspected	51	51	-	MD 22.2 lower (84.35 lower to 39.95 higher)	⊕◯◯◯ VERY LOW	IMPORTANT

CI: confidence interval; MD: mean difference; OR = odds ratio; RR = risk ratio; STS = sodium thiosulphate; UP = uremic pruritus.
